# Influence of the support offered to breastfeeding by maternity hospitals

**DOI:** 10.1590/S0034-8910.2015049005354

**Published:** 2015-12-16

**Authors:** Adriana Passanha, Maria Helena D’Aquino Benício, Sônia Isoyama Venâncio, Márcia Cristina Guerreiro dos Reis

**Affiliations:** I Programa de Pós-Graduação em Nutrição em Saúde Pública. Faculdade de Saúde Pública. Universidade de São Paulo. São Paulo, SP, Brasil; II Departamento de Nutrição. Faculdade de Saúde Pública. Universidade de São Paulo. São Paulo, SP, Brasil; III Núcleo de Evidências. Instituto de Saúde. Secretaria Estadual da Saúde de São Paulo. São Paulo, SP, Brasil; IV Programa de Aleitamento Materno. Secretaria Municipal da Saúde de Ribeirão Preto. Ribeirão Preto, SP, Brasil

**Keywords:** Breast Feeding, Hospitals, Maternity, Maternal-Child Health Services, Baby-Friendly Hospital Initiative

## Abstract

**OBJECTIVE:**

To evaluate whether the support offered by maternity hospitals is associated with higher prevalences of exclusive and predominant breastfeeding.

**METHODS:**

This is a cross-sectional study including a representative sample of 916 infants less than six months who were born in maternity hospitals, in Ribeirao Preto, Sao Paulo, Southeastern Brazil, 2011. The maternity hospitals were evaluated in relation to their fulfillment of the Ten Steps to Successful Breastfeeding. Data were collected regarding breastfeeding patterns, the birth hospital and other characteristics. The individualized effect of the study factor on exclusive and predominant breastfeeding was analyzed using Poisson multiple regression with robust variance.

**RESULTS:**

Predominant breastfeeding tended to be more prevalent when the number of fulfilled steps was higher (*p* of linear trend = 0.057). The step related to not offering artificial teats or pacifiers to breastfed infants and that related to encouraging the establishment of breastfeeding support groups were associated, respectively, to a higher prevalence of exclusive (PR = 1.26; 95%CI 1.04;1.54) and predominant breastfeeding (PR = 1.55; 95%CI 1.01;2.39), after an adjustment was performed for confounding variables.

**CONCLUSIONS:**

We observed a positive association between support offered by maternity hospitals and prevalences of exclusive and predominant breastfeeding. These results can be useful to other locations with similar characteristics (cities with hospitals that fulfill the Ten Steps to Successful Breastfeeding) to provide incentive to breastfeeding, by means of promoting, protecting and supporting breastfeeding in maternity hospitals.

## INTRODUCTION

Breastfeeding is the best way for children to reach their full development.^19,26^ Only breast milk can provide the nutritional and immunological needs as well as see to the infant’s physiological limitations.^26^ However, the frequency of breastfeeding in Brazil is still below the recommendation set out by the World Health Organization (WHO): breast milk to be exclusively offered until six months of age and supplemented with other foods until the baby is two years old or more.^26^ According to the *II Pesquisa de Prevalência de Aleitamento Materno nas Capitais Brasileiras e Distrito Federal* (Second Survey on the Prevalence of Breastfeeding in the Brazilian State Capitals and the Federal District),^17^ the national prevalence of exclusive breastfeeding among children under six months of age is 41.0%. The city of Sao Paulo has a similar prevalence: 39.1%. These percentage levels are classified as “fair” according to the parameters set out by the WHO.^27^

With the objective of protecting, promoting, and supporting breastfeeding in Brazil, several actions have been implemented since 1981, such as: The Kangaroo Mother Care, approval of the Brazilian Norm for Commercialization of Foods for Infants and Young Children, Artificial Teats, Pacifiers and Baby Feeding Bottles, deployment of an extensive network of Human Milk Banks, launching of the *Estratégia Amamenta e Alimenta Brasil* (Brazilian Breastfeeding and Feeding Strategy), and social mobilization actions, such as the World Breastfeeding Week.^25^

Several factors can have a negative influence on breastfeeding, but care provided to women and their children is vital to this practice’s success.^16^ Thus, the WHO and Unicef (United Nations Children’s Fund) launched the Baby-Friendly Hospital Initiative (BFHI) in 1991. If a maternity hospital is to be accredited as a Baby-Friendly Hospital (BFH), the Ten Steps to Successful Breastfeeding must be realized,^12^ which are listed below:

1. Have a written breastfeeding policy that is routinely communicated to all health care staff;
2. Train all health care staff in skills necessary to implement this policy;
3. Inform all pregnant women about the benefits and management of breastfeeding;
4. Help mothers initiate breastfeeding within a half-hour of birth;
5. Show mothers how to breastfeed and how to maintain lactation, even if they should be separated from their infants;
6. Give newborn infants no food or drink other than breast milk, unless medically indicated;
7. Practice rooming-in – allow mothers and infants to remain together – 24 hours a day;
8. Encourage breastfeeding on demand;
9. Give no artificial teats or pacifiers (also called dummies or soothers) to breastfeeding infants;
10. Foster the establishment of breastfeeding support groups and refer mothers to them on discharge from the hospital or clinic.

The BFHI aims to tackle one of the main factors that can be harmful to breastfeeding: health practices that interfere with its success. Despite inappropriate conduct in maternity hospitals not being considered to be solely responsible for the low prevalence of breastfeeding,^19^ and evidence that the BFHI contributes to improving its indices,^14,19,24^ studies are scarce that show the impact of the BFHI on this prevalence at a populational level.^3,6,14^

The objective of this study was to evaluate whether the support offered by maternity hospitals is associated with higher prevalences of exclusive and predominant breastfeeding.

## METHODS

This study is cross-sectional in nature and was performed in two stages: the first (February/2011) all maternity hospitals based in Ribeirao Preto, Sao Paulo, Southeastern Brazil, were evaluated, regarding the fulfilment of the “Ten Steps…”. To achieve the aforementioned, the doctors responsible for the Neonatology Service of each hospital studied were interviewed using the Hospital Self-Appraisal Tool referring to the “Ten Steps…”.^12^ During the second stage (August/2011), there was the *Projeto Amamentação e Municípios *(AMAMUNIC – Breastfeeding and Municipalities Project),^a^ which was performed with the objective of collecting information on breastfeeding patterns and the characteristics of the infants and their mothers. The first stage took place six months before the second to obtain the pattern of breastfeeding infants younger than six months old who were born in maternity hospitals, in Ribeirao Preto, and who were (or not) exposed to the fulfillment of the previously mentioned steps, to avoid temporal biases.

Since 1998, most of the cities in the state of Sao Paulo have had information regarding the breastfeeding patterns of infants under one year of age, which were obtained by the AMAMUNIC Project during the National Polio Vaccination Campaign. The interviews are conducted in the queue by trained personnel.^23^ The questionnaire included close-ended questions about milk consumption, maternal or otherwise, and other foods, referring to the day previous to the research. The use of ‘current status’ is recommended when describing infant feeding practices to minimize potential biases that can arise from the respondent’s memory.^28^ In addition, information regarding the children and their mothers’ characteristics are also obtained, which includes the child’s birth location (municipality and hospital).

The sample size was stipulated at 1,000 children under one year of age from the AMAMUNIC, which made it possible to estimate the prevalence of different events related to the children’s health, with 95.0% certainty and a maximum error margin of ± 3.0%; this precision was expected for events with a prevalence of 50.0%.^9^ To select the sample, the two-stage conglomerate sampling procedure was used: the first stage included a random selection of the vaccination stations, and in the second, the children were randomly selected at each station. The sample was considered equiprobabilistic because all the children had the same probability of belonging to the sample: larger vaccination stations presented a higher likelihood to be selected in the first stage, and children from smaller stations were more likely to be selected in the second stage.^b^

Evaluations were only performed on children under six months of age, who had been born in maternity hospitals in Ribeirao Preto, Sao Paulo. Children who had no information regarding their municipality and place of birth were excluded. A total of 1,755 children under one year of age participated in the AMAMUNIC, 953 of whom were under six months of age. Of these, 37 were excluded because they did not meet the eligibility criteria. Thus, 916 children under six months of age were included in this study.

The city of Ribeirao Preto, Sao Paulo, met the two inclusion criteria for entry into the study: namely, having at least one BFH and having participated in the 2011 AMAMUNIC. This medium-sized city is situated in the northeastern region of the state of Sao Paulo, 313 km from its capital.^c^ In 2011, there were approximately 618 thousand inhabitants, the infant mortality rate was 9.8 and the number of live births was 11,790, with almost all (99.7%) taking place in hospitals.^d,e^

The outcomes of this study were exclusive breastfeeding (EB) – the child only receiving his/her mother’s milk and no other liquids or solids –, and predominant breastfeeding (PB) – the child receiving his/her mother’s milk as the predominant source of nutrition, without receiving any other types of milk or formulas, but being able to receive water or water-based drinks.^28^ The covariates of interest corresponded to the characteristics of the infants: age in full days, sex (male; female), birth weight (< 2,500 g; ≥ 2,500 g), type of delivery (cesarean; vaginal) and for the mothers: maternal age group (< 20 years; 20 to 35 years; ≥ 35 years), maternal parity (primiparous; multiparous), employment status (working outside the home; not working outside the home; on maternity leave), education in years of schooling (≤ 8; 9 to 12; and ≥ 12). The study factor corresponded to the hospital practices to encourage breastfeeding (“Ten Steps ...”). For step 3, only four hospitals that had their own prenatal service or prenatal satellite clinic were evaluated, which is in line with the recommendation set out by the Hospital Self-Appraisal Tool.^12^

The association between the independent variables and each response variable was evaluated by crude analysis using the Chi-square test. The individualized effect of the study factor on each outcome was evaluated by multiple Poisson regression with a robust variance, which was due to this being one of the best alternatives to cross-sectional studies with binary outcomes and to it producing good point and interval estimates of prevalence ratio (PR).^5^ The crude PR values and their respective intervals were presented with 95% confidence interval (95%CI).

The influence of the total number of steps reached was estimated (in tertiles) at each maternity hospital and the influence of each of the fulfilled “Ten Steps...”. The covariates with p < 0.20 in the crude analysis and those that varied by more than 10.0% to the PR of the study factor,^13^ to be introduced into the multiple model, remained as adjustment variables. Variables with more than two categories were introduced into the model in a dummy format. Three multiple models were performed: for Model 1, the influence of the steps was controlled by the age of the child and the maternal age group; in Model 2, the mother’s education level was included; and, in Model 3 (only performed for the PB outcome), the type of delivery was added.

The variables which presented PR values between 0 and 1 were interpreted as factors that decrease the prevalence of the outcomes; PR values of > 1 were interpreted as factors that increase their prevalence.

Data analysis was performed using Stata/SE 11.1 software. The association between the study factor and outcome was considered statistically significant when p < 0.05.

This research project was approved by the Ethics Committee of the Faculdade de Saúde Pública of the Universidade de São Paulo (Process 435/2010) and by the Ribeirao Preto Municipal Secretary of Health (Process 396,991/2011). All mothers gave their verbal consent for the questionnaire to be applied.

## RESULTS

From the seven maternity hospitals located in Ribeirao Preto, three were public and were accredited in the BFHI. In 2011, these locations were responsible for 54.9% of live hospital births.^e^

The number of steps fulfilled in each maternity hospital ranged from 1 to 10, with only one of them having fulfilled all the steps. The mean number of steps fulfilled by all hospitals was six: eight steps for the BFH and three for the non-BFH.

There was a predominance of fulfilling the “Ten Steps…” by the BFH; however, steps 1, 2, 3, 4, 5 and 10 were those least fulfilled. Steps 1, 2 and 4 were fulfilled by the same maternity hospitals; with the same happening with steps 6 and 8.

Table 1 presents the characterization of the study population, the prevalence of EB and PB according to these characteristics and the results of the crude analysis. The percentage of children born with low birth weight was 8.7%. There was predominant proportion of cesarean performed in the studied population (58.9%). Most of the mothers (74.4%) were in the 20 to 35 years old age group. There was a slight predominance of primiparous mothers and mothers who were not working outside the home (51.2% and 51.1%, respectively). Most of the women (54.6%) had spent nine to 11 years in education.

**Table 1:** Proportion of children under exclusive and predominant breastfeeding and the underlying prevalence ratios and confidence intervals according to characteristics of the children and their mothers. Ribeirao Preto, SP, Southeastern Brazil, 2011.

Variable	n	EB (%)	PR	95%CI	p	PB (%)	PR	95%CI	p
Age group of child					**< 0.001** ^**a**^				**0.093** ^**a**^
< 1 month	123	62.2	1			8.5	1		
1 |– 2 months	135	45.5	0.73	0.58;0.92		14.4	1.70	0.78;3.68	
2 |– 3 months	152	38.1	0.61	0.48;0.79		17.7	2.08	0.99;4.34	
3 |– 4 months	185	28.7	0.46	0.35;0.60		25.6	3.01	1.51;5.99	
4 |– 5 months	161	23.6	0.38	0.28;0.52		12.6	1.49	0.67;3.29	
5 |– 6 months	160	11.5	0.18	0.12;0.29		16.2	1.91	0.85;4.30	
Sex					0.358				**0.108**
Male	474	31.8	1			14.0	1		
Female	442	34.7	1.09	0.91;1.31		18.6	1.33	0.94;1.39	
Birth weight					**0.158**				0.326
< 2,500 g	78	26.0	1			11.9	1		
≥ 2,500 g	817	34.1	1.32	0.90;1.95		16.8	1.40	0.71;2.76	
Type of delivery					0.992				
Cesarean	540	33.2	1			12.2	1		**0.001**
Vaginal	376	33.2	1.00	0.91;1.10		22.5	1.84	1.31;2.61	
Maternal age group									
< 20 years	103	22.6	1		**0.044** ^**a**^	34.8	1		**0.002** ^**a**^
20 |– 35 years	188	37.3	1.65	1.14;2.40		14.6	0.42	0.28;0.62	
≥ 35 years	105	35.3	1.57	1.00;2.44		13.3	0.38	0.20;0.72	
Maternal parity					**0.001**				0.701
Primiparous	415	29.6	1			17.3	1		
Multiparous	398	40.9	1.38	1.14;1.68		16.2	0.93	0.65;1.34	
Employment status					**< 0.001** ^**a**^				0.536^a^
Working outside the home	163	23.5	1			15.5	1		
Not working outside the home	415	35.5	1.52	1.12;2.06		18.9	1.22	0.74;2.01	
On maternity leave	234	42.5	1.81	1.32;2.49		13.4	0.90	0.51;1.59	
Education					**0.106** ^**a**^				**0.001** ^**a**^
≤ 8 years of study	189	32.0	1			25.4	1		
9 |– 12 years of study	444	34.5	1.08	0.84;1.38		16.6	0.65	0.44;0.96	
≥ 12 years of study	181	40.2	1.26	0.95;1.66		9.7	0.38	0.21;0.68	

EB: exclusive breastfeeding; PB: predominant breastfeeding

^a^ p of linear trend.

Values of p < 0.20 are presented in bold.

The prevalence of EB and PB in children under six months of age was 33.2% and 16.3% respectively. The percentage of children who had received breast milk in the previous 24 hours was 82.8%. Most of the children (57.6%) had been born in a BFH (data not presented in the table).

We observed a decreasing prevalence of EB along with the increasing age of the children; however, multiparity and the maternity leave showed an increase in the prevalence of this outcome. The increasing age of the mother was associated with a higher prevalence of EB and a lower prevalence of PB. PB was more prevalent among children who had undergone a vaginal birth and less prevalent among children of mothers with higher levels of education (Table 1).

Table 2 shows the prevalence of EB and PB, the fulfillment of the “Ten Steps...” and the results of the crude analysis. The majority of the children had not been exposed to the fulfillment of steps 1, 2, 4 and 5. Generally speaking, the prevalence of EB and PB tended to increase at each fulfilled step. During the analysis using tertiles, the PB was more prevalent along with the increasing number of steps fulfilled. None of the children, who had not been exposed to the fulfilment of steps 6 and 8, were in PB when they were interviewed; therefore, the influence of these steps on this outcome could not be studied.

**Table 2:** Proportion of children under exclusive and predominant breastfeeding and the underlying prevalence ratios and confidence intervals according to the fulfillment of the Ten Steps to Successful Breastfeeding. Ribeirao Preto, SP, Southeastern Brazil, 2011.

Variable	n	EB (%)	PR	95%CI	p	PB (%)	PR	95%CI	p
Steps (tertiles)					0.822^a^				**< 0.001** ^**a**^
1 to 4	371	32.3	1			10.1	1		
5 to 8	315	34.5	1.07	0.86;1.32		18.8	1.87	1.19;2.92	
10	230	32.9	1.02	0.80;1.29		23.5	2.32	1.49;3.65	
Step 1^b^					0.621				**0.011**
No	563	32.6	1			13.4	1		
Yes	353	34.2	1.05	0.87;1.27		20.9	1.56	1.11;2.20	
Step 3^c^					0.527				0.165
No	140	36.0	1			15.8	1		
Yes	405	33.1	0.92	0.70;1.20		22.6	1.43	0.86;2.35	
Step 5					0.657				0.016
No	546	32.7	1			13.4	1		
Yes	370	34.1	1.04	0.86;1.26		20.5	1.53	1.08;2.16	
Step 6^d^					0.388				–
No	14	21.4	1			0.0	–		
Yes	902	33.4	1.56	0.57;4.27		16.5	–		
Step 7					0.113				0.971
No	38	44.4	1			16.0	1		
Yes	878	32.8	0.74	0.51;1.08		16.3	1.02	0.41;2.54	
Step 9					0.639				**< 0.001**
No	371	32.3	1			10.1	1		
Yes	545	33.8	1.05	0.87;1.27		20.8	2.06	1.38;3.09	
Step 10					0.936				**< 0.001**
No	511	33.3	1			11.6	1		
Yes	405	33.1	0.99	0.82;1.20		22.6	1.95	1.37;2.77	

EB: exclusive breastfeeding; PB: predominant breastfeeding

^a^ p of linear trend.

^b ^The results for step 1 are the same as steps 2 and 4.

^c^ In three hospitals, the fulfillment of the step is not applicable.

^d^ The results for step 6 are the same as step 8. These steps could not be evaluated for the PB because all children in PB had been exposed to the fulfillment of steps 6 and 8 when interviewed.

Values of p < 0.05 are presented in bold.

The Figure presents the proportion of children exposed to the fulfillment of each step. Almost all the children were exposed to steps 6, 7 and 8. Steps 1, 2 and 4 had a lower frequency of exposure. A quarter of the study population was exposed to the fulfillment of all the steps.

**Figure:**
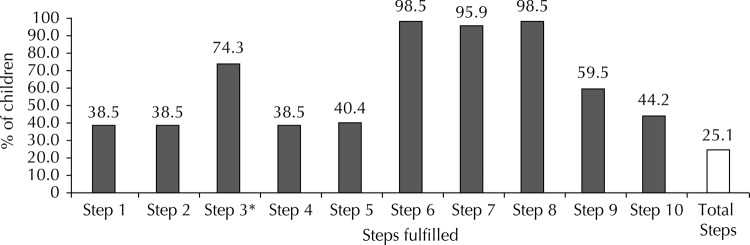
Proportion of children exposed to the fulfillment of the Ten Steps to Successful Breastfeeding. Ribeirao Preto, SP, Southeastern Brazil, 2011.

* In three hospitals, the fulfillment of the step is not applicable.

For the EB outcome, the age of the child, maternal age group and education of the mother met the entry criteria for its incorporation into the multiple model; for the PB, the variables that met these criteria were the same, in addition to the type of delivery.

Table 3 shows the results of the multivariate analysis. For PB, a significant dose-response relationship was observed by its increasing prevalence in line with increasing the number of steps fulfilled in the analysis controlled by age of the child and maternal age group (p = 0.001), and this significance was still present even after maternal education level was included in the model (p = 0.012). When the the type of delivery was added, the significance becomes slightly above the critical level (p = 0.057), albeit with the same increasing trend being observed.

**Table 3:** Prevalence Ratios adjusted for exclusive and predominant breastfeeding according to the fulfillment of the Ten Steps to Successful Breastfeeding. Ribeirao Preto, SP, Southeastern Brazil, 2011.

	EB				PB					

Variable	Model 1^a^		Model 2^b^		Model 1^a^		Model 2^b^		Model 3^c^	

	PR	95%CI	PR	95%CI	PR	95%CI	PR	95%CI	PR	95%CI
Steps (tertiles)	p = 0.424		p = 0.101		**p = 0.001**		**p = 0.012**		p = 0.057	
1 to 4	1		1		1		1		1	
5 to 8	1.18	0.96;1.45	1.30	1.05;1.61	1.61	1.01;2.58	1.38	0.82;2.32	1.27	0.72;2.25
10	1.07	0.85;1.35	1.20	0.94;1.54	2.21	1.39;3.50	1.88	1.12;3.15	1.71	0.94;3.11
Step 1^d^										
No	1		1		1		1		1	
Yes	1.05	0.87;1.26	1.10	0.91;1.34	**1.49**	**1.05;2.11**	1.30	0.89;1.90	1.19	0.80;1.79
Step 3^e^										
No	1		1		1		1		1	
Yes	0.97	0.76;1.25	1.00	0.77;1.30	1.53	0.89;2.63	1.51	0.88;2.59	1.49	0.87;2.55
Step 5										
No	1		1		1		1		1	
Yes	1.04	0.87;1.25	1.09	0.91;1.32	**1.47**	**1.03;2.09**	1.30	0.89;1.88	1.20	0.80;1.78
Step 6^f^										
No	1		1		–		–		–	
Yes	1.20	0.52;2.75	1.26	0.55;2.88	–		–		–	
Step 7										
No	1		1		1		1		1	
Yes	0.77	0.53;1.11	0.80	0.56;1.16	0.82	0.32;2.07	0.68	0.27;1.70	0.66	0.26;1.66
Step 9										
No	1		1		1		1		1	
Yes	1.14	0.94;1.36	**1.26**	**1.04;1.54**	**1.87**	**1.23;2.85**	1.59	0.99;2.54	1.43	0.84;2.45
Step 10										
No	1		1		1		1		1	
Yes	1.08	0.90;1.29	1.18	0.96;1.44	**1.89**	**1.29;2.76**	**1.65**	**1.11;2.47**	**1.55**	**1.01;2.39**

EB: exclusive breastfeeding; PB: predominant breastfeeding

^a^ Model 1: control by age of the child + maternal age group.

^b^ Model 2: Model 1 + maternal education level.

^c ^Model 3: Model 2 + type of birth.

^d^ The results for step 1 are the same as steps 2 and 4.

^e^ In three hospitals, the fulfillment of the step is not applicable.

^f^ The results for step 6 are the same as step 8. These steps could not be evaluated for the PB because all children in PB had been exposed to the fulfillment of steps 6 and 8 when interviewed.

Values of p < 0.05 are presented in bold.

As regards each of the “Ten Steps…”, the fulfillment of step 9 was associated with a significant increase in the prevalence of EB in the analysis adjusted by age of the child, maternal age group and maternal education level (PR = 1.26; 95%CI 1.04;1.54). For PB, the fulfillment of steps 1, 2, 4, 5, 9 and 10 is associated to a high prevalence when the analysis was controlled by the age of the child and maternal age group. When the maternal education variable was included, only fulfilling step 10 presented a significant increase in the prevalence of PB, and this significance remained the same when the type of delivery variable was added to the model (PR = 1.55; 95%CI 1.01;2.39).

## DISCUSSION

Fulfilling a larger number of steps revealed a tendency for the prevalence of PB to increase. Establishing breastfeeding support groups and not offering artificial teats to children being breastfed increased, respectively, PB and EB prevalence in children younger than six months of age.

EB prevalence was only slightly elevated when steps 5 to 8 were fulfilled, and the extent of the effect from the BFHI declined along with the fulfillment of all the 10 steps. However, these differences were not statistically significant.

There was a predominance of fulfilling the “Ten Steps...” by the BFH, which are public in Ribeirão Preto. A study in the city of Sao Paulo compared public and private maternity hospitals and noted that fulfilling all steps tended to be better in public maternity hospitals.^22^ While analyzing compliance among BFH and non-BFH maternity hospitals, a study conducted in Taiwan found better fulfillment among those accredited in the BFHI.^8^ The association between the prevalence of EB and birth in BFH, up to two months of age, was found by a study on 64 Brazilian municipalities, which showed that being born in these maternity hospitals increased the prevalence of EB in this age group by 13.0%.^24^

Not being exposed to any of the “Ten Steps…” could increase the probability of prematurely interrupting breastfeeding by seven times,^11^ in addition, the duration of breastfeeding is positively associated with the total number of steps completed by maternity hospitals.^1,7,8^ The study performed in Taiwan examined the association between number of steps, experienced by 2,079 mothers, and breastfeeding. This study in Taiwan found that only 1.0% of the mothers had been exposed to all the steps, and that the prevalence of the outcome was increased with the increase in the number of practices experienced, after the confounding factors had been controled.^8^ During this present study, 25.1% of the children were exposed to fulfilling all the steps, and the prevalence of PB increased along with the higher the number of steps fulfilled, which shows a positive influence of BFHI on breastfeeding.

The study conducted in Taiwan noted that steps 1, 2, 3 and 5 presented the best fulfillment rates, which is different from this study, but, similarly, it found a low fulfillment of step 4.^8^ During this present study, the same two maternity hospitals fulfilled steps 1, 2 and 4. The low fulfillment of step 4 may indicate little importance being given to the newborn having skin-to-skin contact with his/her mother soon after birth, because even during cesarean this can occur if the hospital staff is well trained and made aware of this importance^3^ – which is directly related to step 2. This relationship can also involve step 1, because hospital staff training is negatively affected when there is a no regulation regarding breastfeeding in maternity hospitals.^12^ Additionally, training the staff is essential so that all the other steps are fulfilled in their entirety, and that mothers receive effective support and guidance for successful breastfeeding.^2^

Findings similar to those found during this study were identified in a study conducted in the Brazilian city of Salvador,^20^ and during a study in the BFH in the Southeastern region of Brazil,^4^ in which, steps 6, 7 and 9 and 6 to 9 were fulfilled, respectively, with greater frequency. During this study, steps 6 and 8 were fulfilled by the same six maternity hospitals. This elevated fulfillment indicates that almost all the maternity hospitals in Ribeirao Preto do not accept donations of breast-milk substitutes from food companies, which promotes exclusive and on demand breastfeeding in the hospital environment.^19^

Not offering artificial teats to breastfeeding children significantly increased the prevalence of EB during this study. In fact, using artificial teats is associated with prematurely ceasing EB^10,15^ and children who use pacifiers may be twice as likely to not experience EB in their first six months of life.^21^ Pacifiers are mainly used to soothe newborns in various situations;^15^ however, sucking on a pacifier is different from that performed on the breast. Using pacifiers causes the phenomenon known as “nipple confusion”, which can decrease breastfeeding frequency and lead the child to premature weaning.^10,15^ Furthermore, using pacifiers is associated with an increased prevalence of using baby feeding bottles,^10^ which is considered the main alternative method for feeding children when mothers cannot successful breastfeed their child; their use also leads to “nipple confusion” and can prematurely interrupt breastfeeding.^15,19^ These findings reinforce the association found during this study between fulfilling step 9 and the increased prevalence of EB, as well as the importance of fulfilling this step.

PB prevalence significantly increased when step 10 was fulfilled. It is a matter for concern that only two maternity hospitals fulfilled step 10, because fostering the establishment of breastfeeding support groups makes it possible for breastfeeding woman to continually receive support, which extends the incentive for exclusive or predominant breastfeeding after the women have been released from hospital.^2,6,19^ In spite of the recommendation for children under six months of age to be under EB, children under PB receive breast milk as their predominant source of nutrition and are not fed with other types of milk or formulas. In addition, practicing PB showed that it has a positive impact on reducing child mortality: a multicenter cohort study conducted with 9,424 children observed that partially breastfed children (who received breast milk and other types of non-human milk) and non-breastfed children, between six and 26 weeks old, were at a higher risk of death (2.5 and 10.5 times, respectively) compared to those children under PB.^4^

It can be said that step 10 is the only one that is not directly related to the hospital sphere of maternity hospitals, because it refers to the establishment of breastfeeding support groups which the mothers must be forwarded to following their discharge from hospital. In this sense, the Ministry of Health is implementing, in addition to the BFHI, new actions for promoting, protecting and supporting breastfeeding, such as the *Estratégia Amamenta e Alimenta Brasil* (Brazilian Breastfeeding and Feeding Strategy), in the basic health care sphere, with the objective of continuing the work of encouraging breastfeeding by maternity hospitals during childbirth hospitalization.^18^

One potential limitation of this study was that evaluating the fulfillment of the “Ten Steps…” was done only by interviewing the doctor responsible for the neonatology service of each maternity hospital, the assumption being that this would be the most appropriate professional to report the situation regarding the care provided to children and their mothers. However, the reliability of the reports can be attributed to the fact that the professionals were aware that the interview would not have any implications regarding the accreditation of these maternity hospitals in the BFHI. Each doctor was made aware of the confidentiality of the information in the study before the interview; they understood that that the hospital’s name would not ever be published.

To avoid the bias related to the temporality associating exposure to the “Ten Steps...” and the prevalence of breastfeeding, the maternity hospitals were evaluated six months before the survey in the vaccination campaign, therefore it was possible to obtain a pattern of breastfeeding of children under six months of age who were born or had received care in hospitals in the city under study and that, with this, we were able to say whether the children had been exposed to the fulfilment of the “Ten Steps...” or not.

The methodology that was adopted for this study can be considered a key differentiator, as it can be easily replicated by cities that monitor infant feeding practices in surveys conducted during vaccination campaigns, since this strategy is complemented by evaluating maternity hospitals in its domain.

In children younger than six months of age, the prevalence of PB showed a tendency to increase when the number of fulfilled steps was higher. During a separate analysis for each step, the fulfillment of each one was observed to have a tendency to increase the prevalence of EB and PB. The steps referring to not offering artificial teats and fostering the establishment of breastfeeding support groups showed, respectively, a positive association with the prevalence of EB and PB. These results can be beneficial to other locations with similar characteristics (cities with hospitals that fulfill the Ten Steps to Successful Breastfeeding) in promoting, protecting and supporting breastfeeding, which is done by means of pro-breastfeeding actions performed by the maternity hospitals. Constantly encouraging the implementation and evaluation of such actions at these sites is necessary to continue the stimulus in improving the prevalences of breastfeeding.

## Footnotes

a The AMAMUNIC Project is a cross-sectional study performed during vaccination campaigns. The cities are contacted annually regarding the proposal to perform the research, with the decision of whether or not to join the project being theirs. The interviewers are trained to perform the investigation in a workshop lasting eight hours, during which they apply the questionnaire to individuals with children under one year of age. After the information has been collected, the data is entered and analyzed using computer software, which, from 2008, was adapted to an online system,which can be accessed through the following link: http://www.redeblh.icict.fiocruz.br/pesqam/

b Instituto de Saúde. Avaliação de práticas alimentares no primeiro ano de vida em dias nacionais de vacinação: manual do Coordenador Municipal. São Paulo (SP): Secretaria do Estado da Saúde; 2010.

c Prefeitura Municipal de Ribeirão Preto. Dados geográficos: Ribeirão Preto (SP); 2012 [cited 2012 Sept 28]. Available from: http://www.ribeiraopreto.sp.gov.br/crp/dados/local/i01localacesso.htm

d Fundação SEADE. Perfil municipal de Ribeirão Preto. São Paulo (SP): Fundação Seade; 2012 [cited 2012 Sept 28]. Available from: http://www.seade.gov.br/produtos/perfil/perfil.php

e Prefeitura Municipal de Ribeirão Preto, Secretaria Municipal da Saúde. Dados referentes a Nascidos Vivos no Município de Ribeirão Preto: Nascidos Vivos - Ribeirão Preto (SP). Ribeirão Preto (SP): Secretaria Municipal da Saúde; 2012 [cited 2012 Sept 28]. Available from: http://www.ribeiraopreto.sp.gov.br/ssaude/vigilancia/vigep/tabnet/i16nascidos.php
