# Supplementary material for: Influence of the support offered to breastfeeding by maternity hospitals
Source: Rev Saude Publica. 2015 Dec 16;49:85. doi: 10.1590/S0034-8910.2015049005354 (PMC4687822; doi:10.1590/S0034-8910.2015049005354)
Supplement: Translation

# Translation: Influência do apoio ao aleitamento materno oferecido pelas maternidades

**Authors:** Adriana Passanha, Maria Helena D’Aquino Benício, Sônia Isoyama Venâncio, Márcia Cristina Guerreiro dos Reis

**Affiliations:** I Programa de Pós-Graduação em Nutrição em Saúde Pública. Faculdade de Saúde Pública. Universidade de São Paulo. São Paulo, SP, Brasil; II Departamento de Nutrição. Faculdade de Saúde Pública. Universidade de São Paulo. São Paulo, SP, Brasil; III Núcleo de Evidências. Instituto de Saúde. Secretaria Estadual da Saúde de São Paulo. São Paulo, SP, Brasil; IV Programa de Aleitamento Materno. Secretaria Municipal da Saúde de Ribeirão Preto. Ribeirão Preto, SP, Brasil

**Keywords:** Aleitamento Materno, Maternidades, Serviços de Saúde Materno-Infantil, Iniciativa Hospital Amigo da Criança

## Abstract

**OBJETIVO:**

Analisar se o apoio oferecido pelas maternidades associa-se à maior prevalência de aleitamento materno exclusivo e predominante.

**MÉTODOS:**

Estudo transversal, com amostra representativa de 916 crianças com menos de seis meses nascidas em maternidades, em Ribeirão Preto, SP, em 2011. As maternidades foram avaliadas em relação ao cumprimento dos Dez Passos para o Sucesso do Aleitamento Materno. Foram coletados dados sobre padrão de amamentação, hospital de nascimento e demais características. O efeito individualizado do fator de estudo sobre o aleitamento materno exclusivo e o predominante foi avaliado mediante análise de regressão múltipla de Poisson, com variância robusta.

**RESULTADOS:**

O aleitamento materno predominante tendeu a ser mais prevalente quanto maior o número de passos cumpridos (p de tendência = 0,057). Os passos relacionados a não oferecer bicos artificiais a crianças amamentadas e a encorajar o estabelecimento de grupos de apoio à amamentação associaram-se, respectivamente, à maior prevalência de aleitamento materno exclusivo (RP = 1,26; IC95% 1,04;1,54) e predominante (RP = 1,55; IC95% 1,01;2,39), após ajuste pelas variáveis de confusão.

**CONCLUSÕES:**

Observou-se associação positiva entre o apoio oferecido pelas maternidades e as prevalências de aleitamento materno exclusivo e predominante. Os resultados trazem subsídios para que localidades com características semelhantes (municípios com hospitais que cumprem os Dez Passos para o Sucesso do Aleitamento Materno) incentivem a amamentação, por meio da promoção, proteção e apoio ao aleitamento materno em maternidades.

## INTRODUÇÃO

A amamentação é a melhor maneira de promover o pleno desenvolvimento infantil.^19,26^ Somente o leite materno atende às necessidades nutricionais e imunológicas e às limitações fisiológicas do lactente.^26^ Entretanto, a prática de amamentação no Brasil ainda está aquém da recomendação da Organização Mundial da Saúde (OMS): leite materno, de modo exclusivo, até os seis meses, e complementado com outros alimentos até os dois anos ou mais.^26^ De acordo com a II Pesquisa de Prevalência de Aleitamento Materno nas Capitais Brasileiras e Distrito Federal,^17^ a prevalência nacional de amamentação exclusiva entre os menores de seis meses é de 41,0%. Na cidade de São Paulo, SP, o quadro é semelhante: 39,1%. Tais valores são classificados como “razoáveis” pelos parâmetros da OMS.^27^

Com o intuito de proteger, promover e apoiar a amamentação, várias ações vêm sendo implementadas no Brasil desde 1981, tais como: Método Canguru, aprovação da Norma Brasileira de Comercialização de Alimentos para Lactentes e Crianças de Primeira Infância, Bicos, Chupetas e Mamadeiras, implantação de ampla rede de Bancos de Leite Humano, lançamento da Estratégia Amamenta e Alimenta Brasil, e ações de mobilização social, como a Semana Mundial de Aleitamento Materno.^25^

Há diversos fatores que podem influenciar negativamente a amamentação, mas os cuidados prestados às mulheres e às crianças são fundamentais para que essa prática seja bem-sucedida.^16^ Com isso, a OMS e o Unicef (Fundo das Nações Unidas para a Infância) lançaram, em 1991, a Iniciativa Hospital Amigo da Criança (IHAC). Para que a maternidade seja credenciada como Hospital Amigo da Criança (HAC), é necessário que sejam cumpridos os Dez Passos para o Sucesso do Aleitamento Materno,^12^ apresentados a seguir:

1. Ter uma política de aleitamento materno escrita que seja rotineiramente transmitida a toda equipe de cuidados de saúde;
2. Capacitar toda a equipe de cuidados de saúde nas práticas necessárias para implementar esta política;
3. Informar todas as gestantes sobre os benefícios e o manejo do aleitamento materno;
4. Ajudar as mães a iniciar o aleitamento materno na primeira meia hora após o nascimento;
5. Mostrar às mães como amamentar e como manter a lactação mesmo se vierem a ser separadas dos filhos;
6. Não oferecer a recém-nascidos bebida ou alimento que não seja o leite materno, a não ser que haja indicação *médica*;
7. Praticar o alojamento conjunto – permitir que mães e recém-nascidos permaneçam juntos – 24 horas por dia;
8. Incentivar o aleitamento materno sob livre demanda;
9. Não oferecer bicos artificiais ou chupetas a crianças amamentadas;
10. Promover a formação de grupos de apoio à amamentação e encaminhar as mães a esses grupos após a alta da maternidade.

A IHAC visa a enfrentar um dos principais fatores que contribuem para prejudicar o aleitamento materno: as práticas de saúde que interferem no seu sucesso. Apesar das condutas inadequadas nas maternidades não serem consideradas as únicas responsáveis pelas baixas prevalências de amamentação,^19^ e das evidências de que a IHAC contribui para melhorar seus índices,^14,19,24^ são escassos os estudos que mostram o impacto da IHAC sobre tais prevalências, em nível populacional.^3,6,14^

O objetivo deste estudo foi analisar se o apoio oferecido pelas maternidades associa-se à maior prevalência de aleitamento materno exclusivo e predominante.

## MÉTODOS

Estudo transversal, realizado em duas etapas: na primeira (fevereiro/2011) foram avaliadas todas as maternidades localizadas em Ribeirão Preto, SP, em relação ao cumprimento dos “Dez Passos...”. Para tanto, foram entrevistados os médicos responsáveis pelo serviço de neonatologia de cada hospital estudado, utilizando o Instrumento de Autoavaliação Hospitalar referente aos “Dez Passos...”.^12^ Na segunda etapa (agosto/2011) foi realizado o Projeto Amamentação e Municípios (AMAMUNIC),^a^ com o objetivo de coletar informações sobre o padrão de amamentação e as características das crianças e de suas mães. A primeira etapa ocorreu seis meses antes da segunda a fim de obter o padrão de aleitamento materno de crianças menores de seis meses que nasceram nas maternidades do município de Ribeirão Preto e que foram (ou não) expostas ao cumprimento dos citados passos, para evitar vieses relacionados à temporalidade.

Desde 1998, grande parte dos municípios do estado de São Paulo possui informações acerca do padrão de amamentação de menores de um ano, obtidas pelo Projeto AMAMUNIC durante a Campanha Nacional de Vacinação contra a Poliomielite. As entrevistas são realizadas, na fila de espera, por pessoas treinadas.^23^ O questionário inclui perguntas fechadas sobre consumo de leite, materno ou não, e de outros alimentos, referentes ao dia anterior à pesquisa. O uso do *current status* é recomendado para descrever práticas de alimentação infantil e minimiza possíveis vieses resultantes da memória do informante.^28^ São obtidas também características das crianças e de suas mães, incluindo local (município e hospital) de nascimento da criança.

O tamanho amostral foi estipulado em 1.000 crianças menores de um ano do AMAMUNIC, que permitiu estimar a prevalência de diferentes eventos relacionados à saúde infantil, com 95,0% de certeza e erro máximo de ± 3,0%; tal precisão era esperada para eventos com prevalência de 50,0%.^9^ Para seleção da amostra, foi usado o procedimento de amostragem por conglomerados, em dois estágios: no primeiro, foram sorteados os postos de vacinação e, no segundo, sorteadas as crianças em cada posto. A amostra é considerada equiprobabilística, pois todas as crianças tiveram a mesma probabilidade de pertencer à amostra: postos maiores apresentaram maior probabilidade de serem sorteados no primeiro estágio, e crianças dos postos menores apresentaram maior probabilidade de serem sorteadas no segundo.^b^

Foram avaliadas somente as crianças menores de seis meses, nascidas em hospitais-maternidade do município de Ribeirão Preto, SP. Foram excluídas as crianças sem informações sobre município e local de nascimento. Participaram do AMAMUNIC 1.755 crianças menores de um ano, das quais 953 eram menores de seis meses. Destas, 37 foram excluídas por não atenderem aos critérios de elegibilidade estabelecidos. Foram, assim, incluídas no estudo, 916 crianças menores de seis meses.

O município de Ribeirão Preto, SP, preencheu os dois critérios de inclusão para entrada no estudo: ter pelo menos um HAC e participar do AMAMUNIC 2011. O município é de médio porte e está situado na região nordeste do estado de São Paulo, a 313 km da capital.^c^ Em 2011, possuía aproximadamente 618 mil habitantes, taxa de mortalidade infantil de 9,8 e número de nascidos vivos de 11.790, sendo a quase totalidade (99,7%) de ocorrência hospitalar.^d,e^

Os desfechos do presente estudo foram aleitamento materno exclusivo (AME) – a criança recebe leite materno e não recebe outros líquidos ou sólidos –, e aleitamento materno predominante (AMP) – a criança recebe leite materno como fonte predominante de nutrição e não recebe outros tipos de leite ou fórmulas, mas pode receber água ou bebidas à base de água.^28^ As covariáveis de interesse corresponderam às características das crianças: idade em dias completos, sexo (masculino; feminino), peso ao nascer (< 2.500 g; ≥ 2.500 g), tipo de parto (cesárea; vaginal) e das mães: faixa etária (< 20 anos; de 20 a 35 anos; ≥ 35 anos), paridade (primípara; multípara), situação de trabalho (trabalha fora; não trabalha fora; está sob licença maternidade), escolaridade em anos de estudo (≤ 8; 9 a 12; e ≥ 12). O fator de estudo correspondeu às práticas hospitalares de incentivo ao aleitamento materno (“Dez Passos...”). O passo 3 foi avaliado apenas nos quatro hospitais que possuíam serviço próprio de pré-natal ou clínica de pré-natal satélite, como recomendado pelo Instrumento de Autoavaliação Hospitalar.^12^

A associação entre as variáveis independentes e cada variável resposta foi avaliada por análise bruta utilizando o teste de Qui-quadrado. O efeito individualizado do fator de estudo sobre cada desfecho foi avaliado por regressão múltipla de Poisson com variância robusta, por ser uma das melhores alternativas para estudos transversais com desfechos binários e por produzir boas estimativas pontuais e intervalares de razão de prevalência (RP).^5^ Foram apresentados os valores de RP brutos e seus respectivos intervalos com 95% de confiança (IC95%).

Foi estimada a influência do número total de passos cumpridos (em tercis) em cada maternidade e a influência de cada um dos “Dez Passos...” cumpridos. As covariáveis com p < 0,20 na análise bruta e que modificaram em mais de 10,0% a RP do fator de estudo,^13^ ao serem introduzidas no modelo múltiplo, permaneceram como variáveis de ajuste. As variáveis com mais de duas categorias foram introduzidas no modelo em formato *dummy*. Foram realizados três modelos múltiplos: no Modelo 1, a influência dos passos foi controlada por idade da criança e faixa etária materna; no Modelo 2, foi incluída a escolaridade da mãe; e, no Modelo 3 (realizado apenas para o desfecho AMP), adicionou-se a variável tipo de parto.

As variáveis que apresentam valores de RP entre 0 e 1 foram interpretadas como fatores que diminuem a prevalência dos desfechos, bem como as RP > 1 foram interpretadas como fatores que aumentam sua prevalência.

A análise dos dados foi realizada no programa Stata/SE 11.1. A associação entre fator de estudo e desfecho foi considerada estatisticamente significante quando p < 0,05.

Este projeto de pesquisa foi aprovado pelo Comitê de Ética da Faculdade de Saúde Pública (Processo 435/2010) e pela Secretaria Municipal da Saúde de Ribeirão Preto (Processo 396.991/2011). As mães deram seu consentimento verbal para a aplicação do questionário.

## RESULTADOS

Das sete maternidades do município de Ribeirão Preto, três eram públicas e estavam credenciadas na IHAC. Estes locais, em 2011, foram responsáveis por 54,9% dos nascimentos vivos de ocorrência hospitalar.^e^

O número de passos cumpridos em cada maternidade variou de 1 a 10, sendo que somente uma delas cumpriu todos os passos. A média de passos cumpridos por todos os hospitais foi seis: oito passos para os HAC e três para os não-HAC.

Houve predomínio do cumprimento dos “Dez Passos...” pelos HAC; entretanto, os passos 1, 2, 3, 4, 5 e 10 foram os menos cumpridos. Os passos 1, 2 e 4 foram cumpridos pelas mesmas maternidades; o mesmo aconteceu com os passos 6 e 8.

A Tabela 1 mostra a caracterização da população estudada, a prevalência de AME e de AMP segundo tais características e os resultados da análise bruta. O percentual de crianças que nasceram com baixo peso foi de 8,7%. O parto cesáreo predominou na população estudada (58,9%). A maior parte das mães (74,4%) encontrava-se na faixa etária de 20 a 35 anos. Houve discreto predomínio de primíparas e de mães que não trabalhavam fora (51,2% e 51,1%, respectivamente). A maioria das mulheres (54,6%) possuía de nove a 11 anos de estudo.

**Tabela 1:** Proporção de crianças em aleitamento materno exclusivo e predominante e respectivas razões de prevalência e intervalos de confiança segundo características das crianças e mães. Ribeirão Preto, SP, 2011.

Variável	n	AME (%)	RP	IC95%	p	AMP (%)	RP	IC95%	p
Faixa etária da criança					**< 0,001** ^**a**^				**0,093** ^**a**^
< 1 mês	123	62,2	1			8,5	1		
1 |– 2 meses	135	45,5	0,73	0,58;0,92		14,4	1,70	0,78;3,68	
2 |– 3 meses	152	38,1	0,61	0,48;0,79		17,7	2,08	0,99;4,34	
3 |– 4 meses	185	28,7	0,46	0,35;0,60		25,6	3,01	1,51;5,99	
4 |– 5 meses	161	23,6	0,38	0,28;0,52		12,6	1,49	0,67;3,29	
5 |– 6 meses	160	11,5	0,18	0,12;0,29		16,2	1,91	0,85;4,30	
Sexo					0,358				**0,108**
Masculino	474	31,8	1			14,0	1		
Feminino	442	34,7	1,09	0,91;1,31		18,6	1,33	0,94;1,39	
Peso ao nascer					**0,158**				0,326
< 2.500 g	78	26,0	1			11,9	1		
≥ 2.500 g	817	34,1	1,32	0,90;1,95		16,8	1,40	0,71;2,76	
Tipo de parto					0,992				
Cesárea	540	33,2	1			12,2	1		**0,001**
Vaginal	376	33,2	1,00	0,91;1,10		22,5	1,84	1,31;2,61	
Faixa etária materna									
< 20 anos	103	22,6	1		**0,044** ^**a**^	34,8	1		**0,002** ^**a**^
20 |– 35 anos	188	37,3	1,65	1,14;2,40		14,6	0,42	0,28;0,62	
≥ 35 anos	105	35,3	1,57	1,00;2,44		13,3	0,38	0,20;0,72	
Paridade					**0,001**				0,701
Primípara	415	29,6	1			17,3	1		
Multípara	398	40,9	1,38	1,14;1,68		16,2	0,93	0,65;1,34	
Situação de trabalho					**< 0,001** ^**a**^				0,536^a^
Trabalha fora	163	23,5	1			15,5	1		
Não trabalha fora	415	35,5	1,52	1,12;2,06		18,9	1,22	0,74;2,01	
Está sob Licença maternidade	234	42,5	1,81	1,32;2,49		13,4	0,90	0,51;1,59	
Escolaridade					**0,106** ^**a**^				**0,001** ^**a**^
≤ 8 anos de estudo	189	32,0	1			25,4	1		
9 |– 12 anos de estudo	444	34,5	1,08	0,84;1,38		16,6	0,65	0,44;0.96	
≥ 12 anos de estudo	181	40,2	1,26	0,95;1,66		9,7	0,38	0,21;0,68	

AME: aleitamento materno exclusivo; AMP: aleitamento materno predominante

^a^ p de tendência linear.

Valores de p < 0,20 apresentados em negrito.

A prevalência de AME e AMP em menores de seis meses foi 33,2% e 16,3%, respectivamente. O percentual de crianças que receberam leite materno nas últimas 24h foi de 82,8%. A maioria das crianças (57,6%) nasceu em HAC (dados não apresentados em tabela).

Observou-se que, conforme aumenta a idade da criança, a prevalência de AME diminui; porém, a multiparidade e a licença-maternidade mostraram aumento da prevalência deste desfecho. O aumento da idade da mãe associou-se a maiores prevalências de AME e menores de AMP. O AMP foi mais prevalente entre crianças que nasceram de parto vaginal e menos prevalente entre crianças de mães com maior escolaridade (Tabela 1).

A Tabela 2 mostra a prevalência de AME e AMP, o cumprimento dos “Dez Passos...” e os resultados da análise bruta. A maioria das crianças não foi exposta ao cumprimento dos passos 1, 2, 4 e 5. De modo geral, a prevalência de AME e de AMP tendeu a aumentar a cada passo cumprido. Na análise por tercis, o AMP foi mais prevalente com o aumento do número de passos cumpridos. Constatou-se que nenhuma criança não-exposta ao cumprimento dos passos 6 e 8 estava em AMP no momento da entrevista; portanto, a influência de tais passos sobre este desfecho não pôde ser estudada.

**Tabela 2:** Proporção de crianças em aleitamento materno exclusivo e predominante e respectivas razões de prevalência e intervalos de confiança segundo cumprimento dos Dez Passos para o Sucesso do Aleitamento Materno. Ribeirão Preto, SP, 2011.

Variável	n	AME (%)	RP	IC95%	p	AMP (%)	RP	IC95%	p
Passos (tercis)					0,822^a^				**< 0,001** ^**a**^
1 a 4	371	32,3	1			10,1	1		
5 a 8	315	34,5	1,07	0,86;1,32		18,8	1,87	1,19;2,92	
10	230	32,9	1,02	0,80;1,29		23,5	2,32	1,49;3,65	
Passo 1^b^					0,621				**0,011**
Não	563	32,6	1			13,4	1		
Sim	353	34,2	1,05	0,87;1,27		20,9	1,56	1,11;2,20	
Passo 3^c^					0,527				0,165
Não	140	36,0	1			15,8	1		
Sim	405	33,1	0,92	0,70;1,20		22,6	1,43	0,86;2,35	
Passo 5					0,657				0,016
Não	546	32,7	1			13,4	1		
Sim	370	34,1	1,04	0,86;1,26		20,5	1,53	1,08;2,16	
Passo 6^d^					0,388				–
Não	14	21,4	1			0,0	–		
Sim	902	33,4	1,56	0,57;4,27		16,5	–		
Passo 7					0,113				0,971
Não	38	44,4	1			16,0	1		
Sim	878	32,8	0,74	0,51;1,08		16,3	1,02	0,41;2,54	
Passo 9					0,639				**< 0,001**
Não	371	32,3	1			10,1	1		
Sim	545	33,8	1,05	0,87;1,27		20,8	2,06	1,38;3,09	
Passo 10					0,936				**< 0,001**
Não	511	33,3	1			11,6	1		
Sim	405	33,1	0,99	0,82;1,20		22,6	1,95	1,37;2,77	

AME: aleitamento materno exclusivo; AMP: aleitamento materno predominante

^a^ p de tendência linear.

^b^ Os resultados para o passo 1 são os mesmos para os passos 2 e 4.

^c^ Em três hospitais, o cumprimento do passo não se aplica.

^d^ Os resultados para o passo 6 são os mesmos para o passo 8. Tais passos não puderam ser avaliados para o desfecho AMP porque todas as crianças em AMP foram expostas ao cumprimento dos passos 6 e 8 no momento da entrevista.

Valores de p < 0,05 apresentados em negrito.

A Figura apresenta a proporção de crianças expostas ao cumprimento de cada passo. Quase todas as crianças foram expostas aos passos 6, 7 e 8. Os passos com menor frequência de exposição foram 1, 2 e 4. Um quarto da população estudada foi exposta ao cumprimento de todos os passos.

**Figura:** Proporção de crianças expostas ao cumprimento dos Dez Passos para o Sucesso do Aleitamento Materno. Ribeirão Preto, SP, 2011.

* Em três hospitais, o cumprimento do passo 3 não se aplica.

Para o desfecho AME, as variáveis idade da criança, faixa etária materna e escolaridade da mãe atenderam aos critérios de entrada para incorporação no modelo múltiplo; no caso do AMP, as variáveis que atenderam a tais critérios foram as mesmas, além do tipo de parto.

A Tabela 3 mostra os resultados da análise múltipla. Para AMP, observou-se relação dose-resposta significativa de aumento da prevalência conforme aumenta o número de passos cumpridos na análise controlada por idade da criança e faixa etária materna (p = 0,001), e a significância se manteve mesmo após a inclusão da escolaridade materna no modelo (p = 0,012). Ao adicionar o tipo de parto, a significância torna-se discretamente superior ao nível crítico (p = 0,057), mas a mesma tendência de aumento é observada.

**Tabela 3:** Razões de Prevalência ajustadas para aleitamento materno exclusivo e predominante segundo cumprimento dos Dez Passos para o Sucesso do Aleitamento Materno. Ribeirão Preto, SP, 2011.

	AME				AMP					

Variável	Modelo 1^a^		Modelo 2^b^		Modelo 1^a^		Modelo 2^b^		Modelo 3^c^	

	RP	IC95%	RP	IC95%	RP	IC95%	RP	IC95%	RP	IC95%
Passos (tercis)	p = 0,424		p = 0,101		**p = 0,001**		**p = 0,012**		p = 0,057	
1 a 4	1		1		1		1		1	
5 a 8	1,18	0,96;1,45	1,30	1,05;1,61	1,61	1,01;2,58	1,38	0,82;2,32	1,27	0,72;2,25
10	1,07	0,85;1,35	1,20	0,94;1,54	2,21	1,39;3,50	1,88	1,12;3,15	1,71	0,94;3,11
Passo 1^d^										
Não	1		1		1		1		1	
Sim	1,05	0,87;1,26	1,10	0,91;1,34	**1,49**	**1,05;2,11**	1,30	0,89;1,90	1,19	0,80;1,79
Passo 3^e^										
Não	1		1		1		1		1	
Sim	0,97	0,76;1,25	1,00	0,77;1,30	1,53	0,89;2,63	1,51	0,88;2,59	1,49	0,87;2,55
Passo 5										
Não	1		1		1		1		1	
Sim	1,04	0,87;1,25	1,09	0,91;1,32	**1,47**	**1,03;2,09**	1,30	0,89;1,88	1,20	0,80;1,78
Passo 6^f^										
Não	1		1		–		–		–	
Sim	1,20	0,52;2,75	1,26	0,55;2,88	–		–		–	
Passo 7										
Não	1		1		1		1		1	
Sim	0,77	0,53;1,11	0,80	0,56;1,16	0,82	0,32;2,07	0,68	0,27;1,70	0,66	0,26;1,66
Passo 9										
Não	1		1		1		1		1	
Sim	1,14	0,94;1,36	**1,26**	**1,04;1,54**	**1,87**	**1,23;2,85**	1,59	0,99;2,54	1,43	0,84;2,45
Passo 10										
Não	1		1		1		1		1	
Sim	1,08	0,90;1,29	1,18	0,96;1,44	**1,89**	**1,29;2,76**	**1,65**	**1,11;2,47**	**1,55**	**1,01;2,39**

AME: aleitamento materno exclusivo; AMP: aleitamento materno predominante

^a^ Modelo 1: controle por idade da criança + faixa etária materna.

^b^ Modelo 2: Modelo 1 + escolaridade materna.

^c ^Modelo 3: Modelo 2 + tipo de parto.

^d^ Os resultados para o passo 1 são os mesmos para os passos 2 e 4.

^e^ Em três hospitais, o cumprimento do passo não se aplica.

^f^ Os resultados para o passo 6 são os mesmos para o passo 8. Tais passos não puderam ser avaliados para o desfecho AMP porque todas as crianças em AMP foram expostas ao cumprimento dos passos 6 e 8 no momento da entrevista.

Valores de p < 0,05 apresentados em negrito.

Em relação a cada um dos “Dez Passos…”, o cumprimento do passo 9 foi associado ao aumento significativo da prevalência de AME na análise ajustada por idade da criança, faixa etária materna e escolaridade materna (RP = 1,26; IC95% 1,04;1,54). No caso do AMP, o cumprimento dos passos 1, 2, 4, 5, 9 e 10 associou-se à elevação da prevalência quando a análise foi controlada por idade da criança e faixa etária materna. Ao ser inserida a variável escolaridade materna, somente o cumprimento do passo 10 apresentou significância no aumento da prevalência de AMP, e esta significância se manteve quando o tipo de parto foi acrescentado no modelo (RP = 1,55; IC95% 1,01;2,39).

## DISCUSSÃO

O cumprimento do maior número de passos mostrou tendência ascendente na prevalência de AMP. Estabelecer grupos de apoio à amamentação e não oferecer bicos artificiais a crianças amamentadas aumentou, respectivamente, as prevalências de AMP e de AME em crianças menores de seis meses.

A prevalência de AME mostrou apenas discreta elevação ao serem cumpridos os passos 5 a 8, e a magnitude do efeito da IHAC diminuiu com o cumprimento de todos os passos. Entretanto, essas diferenças não foram estatisticamente significantes.

Houve predomínio do cumprimento dos “Dez Passos...” pelos HAC, os quais, em Ribeirão Preto, são públicos. Comparando maternidades públicas e privadas, estudo realizado no município de São Paulo observou que o cumprimento de todos os passos tendeu a ser melhor nas maternidades públicas.^22^ Analisando o cumprimento entre maternidades HAC e não-HAC, estudo conduzido em Taiwan constatou melhor cumprimento dentre aquelas credenciadas na IHAC.^8^ A associação entre prevalência de AME e nascimento em HAC até os dois meses de vida foi encontrada por estudo realizado em 64 municípios brasileiros, mostrando que ter nascido nessas maternidades aumentou em 13,0% a prevalência de AME nessa faixa etária.^24^

Não vivenciar nenhum dos “Dez Passos...” pode aumentar em mais de sete vezes a chance de interrupção precoce de amamentação,^11^ e a duração do aleitamento materno apresenta associação positiva com o número total de passos cumpridos pelas maternidades.^1,7,8^ O estudo conduzido em Taiwan examinou a associação entre número de passos vivenciados por 2.079 mães e aleitamento materno e constatou que apenas 1,0% delas esteve exposta a todos os passos, e que a prevalência do desfecho foi maior com o aumento do número de práticas vivenciadas, após controle por fatores de confusão.^8^ No presente estudo, 25,1% das crianças foram expostas ao cumprimento de todos os passos, e a prevalência de AMP aumentou quanto maior o número de passos cumprido, o que mostra a influência positiva da IHAC no aleitamento materno.

Diferentemente deste estudo, o realizado em Taiwan observou que os passos 1, 2, 3 e 5 apresentaram melhores índices de cumprimento, mas, similarmente, encontrou baixo cumprimento do passo 4.^8^ No presente estudo, as mesmas duas maternidades cumpriram os passos 1, 2 e 4. O baixo cumprimento do passo 4 pode indicar pouca importância de colocar o recém-nascido em contato pele a pele com a mãe logo após o parto, pois mesmo nos partos cesáreos esse contato pode ocorrer se a equipe do hospital estiver bem treinada e sensibilizada^3^ – o que está diretamente relacionado ao passo 2. Esta relação também pode envolver o passo 1, pois o treinamento da equipe hospitalar é prejudicado quando não há uma norma sobre amamentação nas maternidades.^12^ Adicionalmente, treinar a equipe é essencial para que todos os passos sejam cumpridos na íntegra, e para que as mães recebam apoio e orientação efetivos para o sucesso do aleitamento materno.^2^

Achados semelhantes aos deste estudo foram identificados em estudo realizado na cidade de Salvador,^20^ e em estudo nos HAC da região Sudeste do Brasil,^4^ nos quais, respectivamente, os passos 6, 7 e 9 e os 6 a 9 foram cumpridos com maior frequência. No presente estudo, os passos 6 e 8 foram cumpridos pelas mesmas seis maternidades. Este elevado cumprimento indica que quase todas as maternidades de Ribeirão Preto não aceitam doação de substitutos do leite materno por empresas alimentícias, o que favorece a amamentação exclusiva e sob livre demanda no ambiente hospitalar.^19^

Não oferecer bicos artificiais a crianças amamentadas aumentou significativamente a prevalência de AME no presente estudo. De fato, o uso de bicos artificiais está associado à interrupção precoce do AME^10,15^ e crianças que usam chupeta podem apresentar o dobro de chance de não estarem em AME aos seis meses de vida.^21^ O uso de chupetas ocorre, principalmente, para acalmar recém-nascidos em diversas situações;^15^ entretanto, a sucção na chupeta é diferente daquela realizada ao seio. Sua utilização ocasiona o fenômeno denominado “confusão de bicos”, a qual pode diminuir a frequência de amamentação e levar a criança ao desmame precoce.^10,15^ Ademais, o uso de chupetas está associado ao aumento da prevalência do uso de mamadeiras,^10^ consideradas o principal método alternativo para alimentação de crianças quando a amamentação não ocorre com sucesso; seu uso também acarreta a “confusão de bicos” e pode interromper a amamentação precocemente.^1,5,19^ Esses achados reforçam a associação encontrada no presente estudo entre cumprimento do passo 9 e aumento da prevalência de AME, e a importância do cumprimento desse passo.

A prevalência de AMP aumentou significativamente quando o passo 10 foi cumprido. O fato de apenas duas maternidades cumprirem o passo 10 é preocupante, pois encorajar o estabelecimento de grupos de apoio à amamentação possibilita apoio contínuo às lactantes, ampliando o incentivo do aleitamento exclusivo ou predominante para além do período de internação hospitalar.^2,6,19^ Apesar de a recomendação para menores de seis meses ser o AME, no AMP as crianças recebem leite materno como fonte predominante de nutrição e não são alimentadas com outros tipos de leite ou fórmulas. Além disso, a prática de AMP mostrou impacto positivo na redução da mortalidade infantil: estudo de coorte multicêntrico realizado com 9.424 crianças observou que, entre seis e 26 semanas de vida, crianças parcialmente amamentadas (as quais receberam leite materno e outros tipos de leite não-humano) e não-amamentadas apresentaram maior risco de morte (2,5 e 10,5 vezes, respectivamente) em comparação àquelas crianças em AMP.^4^

Pode-se dizer que o passo 10 é o único que não está diretamente relacionado à esfera hospitalar das maternidades, por referir-se ao estabelecimento de grupos de apoio à amamentação para os quais as mães devem ser encaminhadas após a alta do hospital. Nesse sentido, o Ministério da Saúde está implementando, além da IHAC, novas ações para a promoção, proteção e apoio à amamentação, como a Estratégia Amamenta e Alimenta Brasil, no âmbito da atenção básica, com o intuito de dar continuidade ao trabalho de incentivo ao aleitamento materno desempenhado pelas maternidades durante a internação hospitalar para realização do parto.^18^

Uma potencial limitação deste estudo foi a avaliação do cumprimento dos “Dez Passos...” entrevistando-se somente o médico responsável pelo serviço de neonatologia de cada maternidade, assumindo-se que este seria o profissional mais indicado para relatar a situação da atenção prestada às crianças e às mães. Porém, a fidedignidade dos relatos pode ser atribuída ao fato de os profissionais estarem cientes de que a entrevista não traria implicações em relação ao credenciamento das maternidades na IHAC. Cada médico foi informado em relação ao sigilo das informações do estudo antes da realização da entrevista, estando cientes que o nome do hospital não seria divulgado em momento algum.

Para evitar o viés relacionado à temporalidade da associação entre exposição aos “Dez Passos...” e as prevalências de aleitamento materno, as maternidades foram avaliadas seis meses antes da realização do inquérito na campanha de vacinação, sendo possível captar o padrão de aleitamento materno das crianças menores de seis meses que nasceram ou receberam cuidados nos hospitais do município estudado e que, com isso, foram ou não expostas ao cumprimento dos “Dez Passos...”.

A metodologia adotada neste estudo pode ser considerada importante diferencial, pois pode ser facilmente replicada por municípios que adotam o monitoramento das práticas de alimentação infantil em inquéritos realizados nas campanhas de vacinação, desde que essa estratégia seja complementada pela avaliação das maternidades de sua abrangência.

Em crianças menores de seis meses, a prevalência de AMP mostrou tendência ao aumento quanto maior o número de passos cumpridos. Na análise em separado para cada passo, observou-se que o cumprimento de cada um tende a aumentar as prevalências de AME e de AMP. Os passos referentes a não oferecer bicos artificiais e encorajar o estabelecimento de grupos de apoio à amamentação mostraram, respectivamente, associação positiva com as prevalências de AME e de AMP. Tais resultados podem servir como subsídios para outras localidades com características semelhantes (municípios com hospitais que cumprem os Dez Passos para o Sucesso do Aleitamento Materno) promoverem, protegerem e apoiarem o aleitamento materno, por meio de ações pró-amamentação realizadas pelas maternidades. Faz-se necessário incentivar constantemente a implementação e a avaliação de tais ações nesses locais, para o estímulo contínuo na melhoria das prevalências do aleitamento materno.

## Footnotes

a O Projeto AMAMUNIC é um estudo transversal, realizado durante campanhas de vacinação. Os municípios são comunicados anualmente sobre a proposta de realização da pesquisa, sendo espontânea a adesão ao projeto. Os entrevistadores são treinados em uma oficina com duração de oito horas para realização do inquérito, e aplicam o questionário a acompanhantes de crianças menores de um ano. Após a coleta das informações, os dados são digitados e analisados em um aplicativo informatizado, o qual, a partir de 2008, foi adaptado para um sistema *on-line*, sendo possível acessá-lo pelo *link*: http://www.redeblh.icict.fiocruz.br/pesqam/

b Instituto de Saúde. Avaliação de práticas alimentares no primeiro ano de vida em dias nacionais de vacinação: manual do Coordenador Municipal. São Paulo (SP): Secretaria do Estado da Saúde; 2010.

c Prefeitura Municipal de Ribeirão Preto. Dados geográficos: Ribeirão Preto (SP); 2012 [citado 2012 set 28]. Disponível em: http://www.ribeiraopreto.sp.gov.br/crp/dados/local/i01localacesso.htm

d Fundação SEADE. Perfil municipal de Ribeirão Preto. São Paulo (SP): Fundação Seade; 2012 [citado 2012 set 28]. Disponível em: http://www.seade.gov.br/produtos/perfil/perfil.php

e Prefeitura Municipal de Ribeirão Preto, Secretaria Municipal da Saúde. Dados referentes a Nascidos Vivos no Município de Ribeirão Preto: Nascidos Vivos - Ribeirão Preto (SP). Ribeirão Preto (SP): Secretaria Municipal da Saúde; 2012 [citado 2012 set 28]. Disponível em: http://www.ribeiraopreto.sp.gov.br/ssaude/vigilancia/vigep/tabnet/i16nascidos.php

## DESTAQUES

Em 1991, a Organização Mundial da Saúde e o Fundo das Nações Unidas para a Infância lançaram a Iniciativa Hospital Amigo da Criança. Para que a maternidade seja credenciada na Iniciativa, é necessário que cumpra os Dez Passos para o Sucesso do Aleitamento Materno. O presente estudo buscou avaliar se o apoio oferecido pelas maternidades está associado a maiores prevalências de aleitamento materno exclusivo e predominante.

Observou-se que o aleitamento materno predominante tende a ser mais prevalente com o cumprimento de maior número de passos. O passo referente ao não oferecimento de bicos artificiais a crianças amamentadas e aquele relacionado ao encaminhamento das mães a grupos de apoio à amamentação por ocasião da alta hospitalar aumentaram, respectivamente, as prevalências de aleitamento materno exclusivo e predominante.

Práticas inadequadas nos hospitais não são as únicas responsáveis pelos baixos índices de aleitamento materno, mas cuidados apropriados podem constituir um importante requisito para o aumento desses índices. Assim, os resultados do presente estudo mostram a relevância da iniciativa e do cumprimento dos “Dez Passos...”, devido a seus impactos sobre o aumento das prevalências de amamentação exclusiva e predominante em crianças menores de seis meses.

Rita de Cássia Barradas Barata

Editora Científica
